# Postoperative Pain After Root Canal Filling with Different Bioceramic Endodontic Sealers: A Double-Blind Randomized Clinical Pilot Trial

**DOI:** 10.3390/healthcare14152305

**Published:** 2026-07-31

**Authors:** Khalid Alrashedi, Mohammed S. Alzahrani

**Affiliations:** Restorative Dental Sciences Department, Faculty of Dentistry, Al-Baha University, Al-Baha 65779, Saudi Arabia; khalrashidi@bu.edu.sa

**Keywords:** postoperative pain, bioceramic sealer, Bio-C Sealer, TotalFill BC Sealer, root canal treatment, obturation, randomized clinical trial

## Abstract

**Background/Objectives**: Postoperative pain is one of the most frequently reported complications following endodontic treatment. Bioceramic sealers have gained widespread clinical acceptance. However, comparative clinical data on postoperative pain between specific bioceramic sealers remains limited. This double-blind randomized clinical pilot trial aimed to evaluate and compare the incidence and intensity of postoperative pain following root canal obturation using two bioceramic sealers—Bio-C Sealer (Angelus, Brazil) and TotalFill BC Sealer (FKG Dentaire, Switzerland)—over a seven-day follow-up period. **Methods**: A total of 30 patients requiring non-surgical root canal treatment were recruited from the dental clinics at Al-Baha University, Saudi Arabia, and randomly allocated into two equal groups (*n* = 15 per group). All root canals were prepared and then obturated using the tested sealers with the single-cone technique. Postoperative pain was assessed using the Visual Analog Scale (VAS) at 24 h, 48 h, 72 h, and 7 days post-treatment. Statistical analysis was performed using Fisher’s exact test, Chi-square tests, and the independent *t*-test, with a significance threshold set at *p* < 0.05. **Results:** At 24 h, postoperative pain was reported by 33.3% of patients in the Bio-C Sealer group and 26.7% in the TotalFill BC Sealer group, with no statistically significant difference (*p* = 1.000). By 48 h, all patients in the TotalFill BC Sealer group were completely asymptomatic, whereas 13.3% of patients in the Bio-C Sealer group continued to report pain (*p* = 0.483). No significant associations were identified between postoperative pain and gender, tooth type, sealer extrusion, or preoperative pain status in either group (*p* > 0.05). **Conclusions:** Within the limitations of this randomized clinical pilot trial, the preliminary data suggest similar short-term clinical performance between Bio-C Sealer and TotalFill BC Sealer in terms of both the incidence and intensity of postoperative pain following non-surgical root canal treatment.

## 1. Introduction

Pain is one of the most common side effects of endodontic therapy and is described as “an unpleasant psychological and sensory experience associated with or suggested by actual or probable tissue injury” [1]. Its occurrence is influenced by multiple factors, including the host’s immune response, presence of infection, physical trauma, accuracy of working length, apical foramen enlargement, number of treatment visits, analgesic use, gender, filing technique, bacterial extrusion, and type of sealer used during obturation, especially in cases of extrusion, potentially causing a localized inflammatory response [2,3,4,5,6]. The prevalence of postoperative pain following endodontic treatment ranges from 3% to 58% [7], with a significantly higher incidence in mandibular molars (6%) than in maxillary molars (2.2%) [8].

Endodontic treatment involves three key steps: access cavity preparation, cleaning and shaping, and obturation. The obturation phase comprises two essential components: a gutta-percha cone and a sealer [9]. Given the significance of sealers, numerous studies have been conducted to assess their effect on post-treatment pain [10,11]. Bioceramic (calcium silicate-based) sealers have gained increasing popularity in endodontic treatment due to their favorable biological, physical, and clinical properties, such as biocompatibility [10], good flowability [11], and high antibacterial activity and strong adhesion to dentinal walls [12,13,14,15]. Newly developed examples of bioceramic sealers are TotalFill^®^ BC (FKG Dentaire SA, La-Chaux-de-fonds, Switzerland) and Bio-C Sealer (Angelus, Londrína, PR, Brazil). TotalFill BC Sealer is an established, premixed calcium silicate-based sealer with widely documented clinical success, whereas Bio-C Sealer is a more recently introduced, ready-to-use bioceramic formulation featuring distinct physical properties (such as variations in radiopacity and film thickness). These two sealers have been shown to have the same chemical composition but different physical properties such as solubility and setting time. These differences might have an impact on their clinical performance and cause different host reactions [16].

Pain is a highly subjective experience; therefore, the method used for its assessment is critical [17,18]. In the present study, as in many previous investigations, the Visual Analog Scale (VAS) was used to assess postoperative pain. The VAS allows patients to quantify their pain intensity on a scale from 0 to 10, providing a reliable and sensitive method for pain evaluation [19].

According to the literature, there is a lack of in vivo studies on these sealers. None has compared TotalFill BC Sealer with Bio-C Sealer in terms of the postoperative pain that occurs after their use in root canal treatment. Considering the findings of previous studies and the potential influence of obturation materials on postoperative pain, this clinical study was designed to evaluate the effects of TotalFill^®^ BC Sealer and Bio-C Sealer on post-obturation pain. The null hypothesis was that there would be no significant difference in the incidence or intensity of post-obturation pain between the two sealers.

## 2. Materials and Methods

### 2.1. Ethical Considerations

This study was approved by the Research Ethics Committee of Al-Baha University (Ethics No: 2025/46113615) and conformed to the Helsinki Declaration of 1975.

### 2.2. Trial Registration Information

Registry Name: ClinicalTrials.gov.

Registration Number: NCT06891411.

Registration Site: https://clinicaltrials.gov.

### 2.3. Trial Design Framework

This study was conducted as a parallel-group, active-controlled, prospective, double-blind, randomized clinical trial with a 1:1 allocation ratio, strictly satisfying the updated baseline reporting standards of the CONSORT 2025 statement.

### 2.4. Sample Size Estimation

To determine the appropriate sample size for this parallel pilot trial framework, an a priori calculation was performed using G* Power software (version 3.1.9.7; Heinrich-Heine-Universität Düsseldorf, Düsseldorf, Germany) based on continuous primary outcome variance markers derived from related hydraulic sealer clinical literature [10,15].

Anticipating a standard attrition rate due to dependency on phone follow-up, a 10% dropout ratio was accounted for. The mathematical calculation required 13 participants per group. Factoring in potential loss to follow-up, the final sample size was rounded up to 15 participants per intervention arm (*n* = 30 total), which provides sufficient sensitivity to discover clinically meaningful differences in pain levels while preserving statistical validity.

### 2.5. Patient Selection

Patients aged 18 years or older who were medically healthy, classified as American Society of Anesthesiologists (ASA) Physical Status I or II, and required non-surgical root canal treatment were recruited from the dental clinics at Al-Baha University, Saudi Arabia. Over a 6-month period (January–June 2025), all eligible patients, according to the inclusion and exclusion criteria shown in Table 1, received detailed oral and written information about the study and provided written informed consent prior to enrolment.

### 2.6. Baseline Variables and Diagnostic Criteria

To ensure a robust assessment of potential confounding factors affecting postoperative pain outcomes, the following clinical and procedural variables were recorded at baseline and during the treatment phase:

**Tooth Type:** Categorized broadly into molar and non-molar (incisors, canines, and premolars) to evaluate the impact of anatomical complexity.

**Preoperative Pain Status:** Evaluated subjectively by the patient and recorded as either present or absent immediately prior to initiating treatment.

**Lesion Size and Periapical Status:** Determined using periapical radiographs. Periapical status was classified based on the presence or absence of a periapical radiolucency, with lesion size measured horizontally and vertically using digital software and categorized as normal periapical appearance or periapical lesion < 2 mm (per inclusion criteria).

**Sealer Extrusion (“Sealer Puff”):** Assessed via the immediate post-obturation final radiograph and recorded as a binary variable (considered present if any sealer was visible beyond the apical foramen or absent if confined within the root canal system).

### 2.7. Pulpal and Periapical Diagnostic Protocols

Pulpal status was definitively established using a combination of clinical history, visual inspection, and sensibility testing using thermal cold testing (Endo-Ice, Coltene/Whaledent, Cuyahoga Falls, OH, USA). Periapical status was confirmed via digital periapical radiography coupled with clinical percussion and palpation testing. Diagnostic distributions (e.g., symptomatic/asymptomatic irreversible pulpitis or pulp necrosis, paired with normal periapical tissues or symptomatic/asymptomatic apical periodontitis) were tracked to ensure a homogeneous distribution between the Bio-C Sealer and TotalFill BC Sealer intervention groups during data analysis.

### 2.8. Randomization and Blinding (CONSORT Compliance)

**Figure 1:**
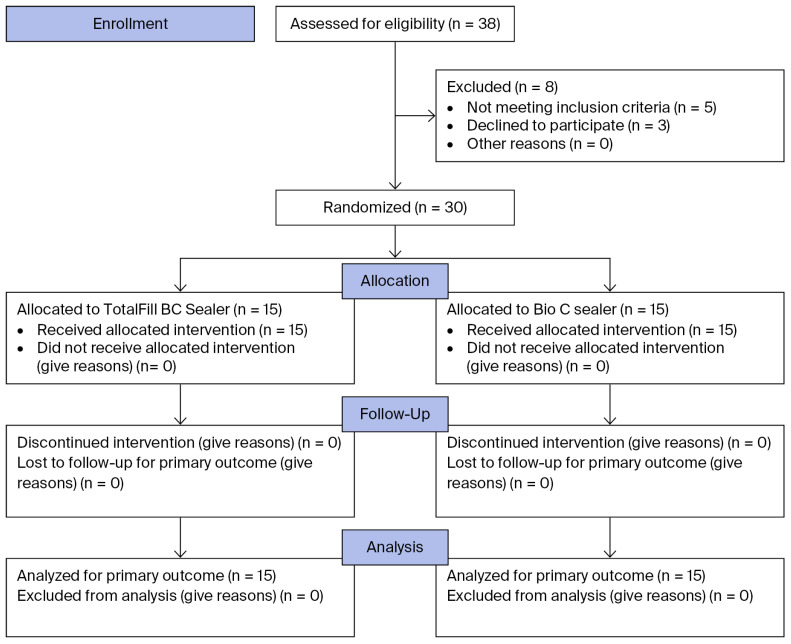
CONSORT 2025 flow diagram illustrating the parallel, double-blind, randomized clinical pilot trial. A total of 30 patients were allocated (1:1), followed up for 7 days, and analyzed, with no attrition.

After obtaining informed consent, participants were randomized using a simple random sampling technique into a parallel-group design. The random allocation sequence was generated using computer-generated random numbers/randomization software. Allocation was performed at a 1:1 ratio, ensuring an equal distribution of patients between the two experimental groups (*n* = 15 per group). Allocation concealment was achieved using sequentially numbered, opaque, sealed envelopes. This study adhered to the standard guidelines for randomized clinical trials (CONSORT Statement 2025). The complete flow of participants through the trial is detailed in Figure 1. Patients were blinded to the treatment allocation, and the clinician was blinded to the sealer allocation. To ensure clinician blinding, the sealers were prepared by a secondary assistant according to the manufacturer’s instructions and provided in a manner that masked the brand identity. This assistant prepared and transferred the assigned sealers into identical, unbranded delivery syringes outside the main clinic room. Consequently, the operating endodontist, the patient, and the independent data collector (who conducted telephone follow-ups) remained strictly blinded.

### 2.9. Treatment Protocol

After confirming the pulpal and periapical diagnosis, preoperative pain was rec-orded via the 10 cm VAS immediately before treatment. Then, the participants were anesthetized with 4% articaine hydrochloride containing 1:100,000 adrenalin. Thereaf-ter, a rubber dam was placed, and the pulp chamber was reached using a diamond bur, which was then copiously irrigated with 5 mL of 5.25% sodium hypochlorite (NaOCl). Treatment was provided with the aid of a dental operating microscope (Zeiss OPMI Pico and Zeiss Extaro 200; Carl Zeiss Meditec AG, Jena, Germany). The working length was established by #10 or larger K-files (Dentsply Maillefer, Ballaigues, Switzerland) and a Root ZX II apex locator (JMorita, Kyoto, Japan) and confirmed on an X-ray. A glide path was created using Pathfiles (Dentsply Maillefer; Ballaigues, Switzerland). The root canals were shaped using the ProTaper Gold system (Dentsply Maillefer; Ballaigues, Switzer-land) which features the exact same geometry and sequence as the classic ProTaper Universal system but introduces proprietary gold-wire metallurgy. The files were used in an in-and-out movement. After each insertion of the file, the canal was again irrigated with NaOCl, and canal patency was checked with a size 10 K-file. Each root canal was irrigated with the same volume of NaOCl and 17% ethylenediaminetetraacetic acid (EDTA, 5 mL), followed by a final irrigation of 5 mL of NaOCl interspersed with normal saline rinses. A plastic device (Easy Clean, Easy Equipamentos Odontológicos, Belo Horizonte, Brazil) was used in reciprocating motion in three sessions of 20 s each to ac-tivate separately and sequentially both NaOCl and EDTA during the final irrigation. The canals were subsequently dried using sterile paper points, and a master gutta-percha cone corresponding to the final instrumentation size was selected. After radiographic verification of the fit, the gutta-percha tip was adjusted with a blade where necessary to ensure precise apical seating. At this stage, one of the two randomized bioceramic seal-ers—Bio-C Sealer or TotalFill BC Sealer—was selected. Prior to beginning the obturation phase during the second appointment, all teeth were clinically verified to be entirely asymptomatic, exhibiting no tenderness to percussion, no sensitivity to palpation, and no persistent localized swelling. The canals were then obturated using a single-cone technique, with the sealer delivered directly into the canal via the manufactur-er-provided syringe tip. Subsequently, the teeth were restored with composite resin, and the occlusion was verified. A final radiograph was obtained to assess and record any in-stances of sealer extrusion. All root canal treatments were performed between January and June 2025 by a single experienced endodontist.Bio-C Sealer was utilized as an active control group to provide a comparative baseline against TotalFill BC Sealer. Given that both materials share a bioceramic base, this comparison allowed for an evaluation of clinical outcomes—specifically postoperative pain—within the same class of bioactive materials, ensuring that the results are relevant to modern endodontic standards.

Participants were instructed to take either 500 mg of Paracetamol or 600 mg of Ibuprofen if needed for pain relief. Analgesic intake was systematically tracked as a secondary clinical outcome; the absolute necessity for rescue medication (yes/no) and the total number of tablets consumed were recorded on the patient’s diary card, which was returned at the one-week follow-up appointment. The total number of tablets consumed was recorded, and the VAS cards were returned at the one-week follow-up appointment.

Postoperatively, the primary outcome—patient perception of pain—was monitored by a clinician (K.A.), who contacted the participants via telephone to track their progress and remind them to complete the Visual Analog Scale (VAS) card. This scale ranged from 0 (asymptomatic) to 10 (unbearable pain), with scores categorized as none (score of 0), mild pain (scores of 1 and 2), moderate pain (scores of 3–7), or severe pain (scores of 8–10) at 24-h, 48-h, 72-h, and 1-week intervals.

### 2.10. Statistical Analysis

Statistical analysis was performed using SPSS software (Version 25; IBM, Chicago, IL, USA), with the significance threshold set a priori at *p* < 0.05. The baseline homogeneity of the demographic and clinical characteristics of the two groups was analyzed using Chi-square tests, Fisher’s exact test, and the independent *t*-test where appropriate. Because postoperative pain was measured repeatedly within the same participants over four distinct follow-up intervals, these longitudinal observations were naturally correlated. However, due to the exploratory and pilot nature of this trial (*n* = 15 per group), the study was not sufficiently powered to reliably fit complex, multi-parameter longitudinal statistical framework models—such as generalized estimating equations (GEEs) or repeated-measures mixed-effects models—without a substantial risk of statistical overfitting. Consequently, a pragmatic, time point-specific analytical strategy was adopted.

To ensure a comprehensive and robust evaluation of post-obturation discomfort, postoperative pain was assessed using a dual-metric approach incorporating both the continuous Visual Analog Scale (VAS) and a categorical pain scale. The 10 cm VAS serves as a highly sensitive, continuous spatial measurement that captures subtle, millimetric fluctuations in pain intensity with high granularity, providing the interval data necessary for precise parametric statistical analyses of longitudinal pain trajectories. Conversely, categorizing these scores into distinct clinical brackets (none, mild, moderate, and severe) translates abstract numerical data into clinically meaningful thresholds of patient suffering. This dual-pronged assessment bridges the gap between scientific, statistical precision and real-world clinical significance, allowing for the detection of subtle sub-clinical trends while simultaneously presenting clear, practical outcomes regarding the proportion of patients achieving complete, asymptomatic recovery.

## 3. Results

### 3.1. Baseline Demographic and Clinical Characteristics

A total of 30 patients were successfully randomized into two equal groups: the Bio-C Sealer group (*n* = 15) and the TotalFill BC Sealer group (*n* = 15). Statistical analysis confirmed that the treatment cohorts were homogeneous at baseline across all demographic and clinical variables (*p* > 0.05), ensuring that the groups were comparable for the primary outcome assessment. The mean age of the study population was 38.30 ± 16.3 years, with no significant difference between the Bio-C Sealer (34.13 ± 14.2 years) and TotalFill BC Sealer groups (41.87 ± 17.9 years; *p* = 0.201). The gender distribution was balanced, with 50% of the sample being male (*p* = 0.465). Clinical factors—including preoperative pain status (*p* = 0.682), the presence of preoperative swelling (*p* = 1.000), and the size of periapical lesions (*p* = 0.420)—were evenly distributed between the groups. Furthermore, procedural variables such as tooth type (63.3% molars; *p* = 0.705) and the incidence of unintentional sealer extrusion (“sealer puff”), which occurred in 73.3% of cases in both groups (*p* = 1.000), showed no significant intergroup differences.

### 3.2. Participant Flow Diagram

The screening, allocation, and complete progression of participants through the trial phases are illustrated below, adhering fully to the CONSORT 2025 standard reporting guidelines.

### 3.3. Postoperative Pain Assessment

Postoperative pain was evaluated at 24 h, 48 h, 72 h, and 7 days. At 24 h post-treatment, pain was reported by five patients (33.3%) in the Bio-C Sealer group (four with mild pain and one with severe pain) and by four patients (26.7%) in the TotalFill BC Sealer group (all with mild pain), with no statistically significant difference between the groups (*p* = 1.000). By the 48 h evaluation, a notable clinical divergence was observed: all fifteen patients (100%) in the TotalFill BC Sealer group reported complete resolution of symptoms, whereas two patients (13.3%) in the Bio-C Sealer group continued to experience pain (one mild and one severe) (*p* = 0.483). At the 72 h follow-up, severe pain persisted in one patient (6.7%) in the Bio-C Sealer group; however, the severity decreased to mild pain by the 7-day evaluation, while the TotalFill BC Sealer group remained entirely pain-free (*p* = 1.000). Although the TotalFill BC Sealer group reached an asymptomatic state faster, the differences at these intervals did not reach statistical significance (Table 2).

### 3.4. Correlation and Secondary Analyses

A secondary analysis was performed to determine whether clinical or procedural factors influenced the development of postoperative pain. No significant correlation was found between the postoperative pain and the size of the preoperative periapical lesion (*p* = 0.546). Similarly, the presence of a “sealer puff” did not significantly correlate with the incidence of postoperative pain (*p* = 0.732). Exploratory subgroup analyses based on baseline demographics and clinical sub-categories showed no significant associations with postoperative pain scores; however, due to the limited number of patients within these individual subsets, these findings lack definitive statistical reliability. The complete datasets for these secondary subgroup analyses can be found in the online Supplementary File (see Supplementary Tables S1 and S2).

**Figure 2:**
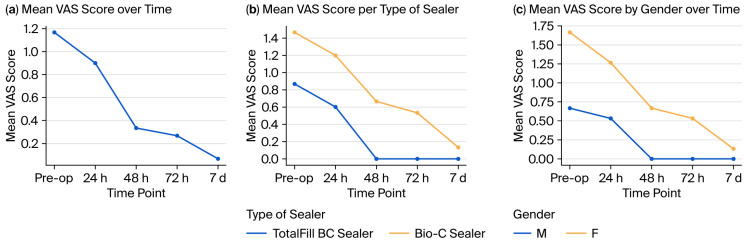
Visual Analog Scale (VAS) pain score trends. (**a**) Overall mean VAS scores across the entire patient cohort over time. (**b**) Comparison of mean VAS scores stratified by sealer type. (**c**) Progression of mean VAS scores stratified by patient gender (M: male; F: female) over the evaluated follow-up periods (Pre-op: preoperative; 24h: 24 h; 48h: 48 h; 72h: 72 h; 7d: 7 days).

While both groups exhibited postoperative pain at 24 h (*p* = 1.000), the TotalFill BC Sealer group showed a more rapid relief of symptoms, achieving a 0% incidence of pain by the 48 h mark and remaining asymptomatic throughout the 7-day follow-up. In contrast, one patient (6.7%) in the Bio-C Sealer group showed lingering postoperative pain symptoms up until day 7 (Figure 2).

## 4. Discussion

Post-endodontic pain is one of the most common complaints reported by patients following root canal therapy and is generally associated with the activation of an inflammatory response in the periapical tissues [20]. This discomfort may negatively affect patients’ daily activities and overall quality of life. Therefore, the present study was conducted to evaluate postoperative pain following endodontic treatment using two different bioceramic sealers: Bio-C Sealer and TotalFill BC Sealer. Furthermore, post-treatment pain is a multifactorial phenomenon. Therefore, to minimize confounding factors, only asymptomatic teeth at the time of obturation were included in the study. Moreover, to ensure standardization during chemomechanical preparation, the same NiTi rotary file system was used for all teeth included in the study. In addition, all procedures were performed by a single operator, and the chemomechanical preparation followed an identical irrigation protocol for every case, thereby minimizing the influence of procedural variables on the study outcomes.

The results of the present study show that postoperative pain was absent in 70% of patients, whereas 30% experienced pain following root canal obturation. These findings are comparable to those of previous studies that reported postoperative pain incidence ranging from 3% to 58% [7,20,21,22,23,24]. In the current study, postoperative pain occurred predominantly within the first 24 h (30% of patients) and decreased markedly over time, reaching only 3.3% by day 7 after primary endodontic treatment. This pattern is consistent with that in previous reports indicating that postoperative pain is most frequent during the early post-treatment period and gradually diminishes over time [10,20,21,22,23,24].

A crucial consideration when interpreting these data is that the recurrent observation of “no statistically significant differences” between Bio-C Sealer and TotalFill BC Sealer must not be misconstrued as definitive clinical evidence of therapeutic equivalence. Because this study was carried out as a small-scale clinical pilot trial (*n* = 15 per group), it has a heightened susceptibility to a Type II statistical error (false negative). The smaller sample size limits the statistical power required to detect subtle clinical differences in pain recovery trajectories or intensity drops between the groups. While the TotalFill BC Sealer group reached an entirely asymptomatic state faster (0% pain at 48 h vs. 13.3% in the Bio-C group), the trial was underpowered to confirm whether this faster resolution constitutes a true therapeutic divergence or a mere random variation. Consequently, these data must be viewed strictly as exploratory and trend-generating.

The incidence of unintentional sealer extrusion (“sealer puff”) observed in this trial was remarkably high, reaching 73.3% in both experimental groups. Rather than reflecting operator error, this high rate is closely linked to the distinct physicochemical and rheological properties of modern hydraulic calcium silicate-based sealers [15]. Premixed bioceramic sealers exhibit low film thickness, high flowability, and hydrophilic properties, allowing them to adapt closely to the intricate root canal anatomy and easily escape through the apical foramen when paired with a single-cone gutta-percha obturation technique. While this high extrusion rate presented a clinical challenge in observing periapical responses, it introduces an important caveat regarding the external validity of our findings. The fact that pain incidence did not significantly correlate with extrusion (*p* = 0.732) might indicate that these bioceramic materials are highly biocompatible in vivo, but clinicians should remain cautious when extrapolating these high extrusion rates to alternative obturation protocols or different operator skill levels.

To better understand the biological mechanisms underlying these findings, it is helpful to examine the underlying material science. Both TotalFill BC Sealer and Bio-C Sealer consist of a calcium silicate core, but they display subtle differences in their formulations, setting kinetics, and trace components [13]. TotalFill BC Sealer is a highly stable, premixed paste that relies entirely on dentinal tubule moisture to initiate its setting reaction, leading to consistent ion release over an extended period. Bio-C Sealer is a ready-to-use formulation that features a modified particle size distribution designed to optimize flow and radiopacity while altering its ultimate solubility and setting speed [16].

In laboratory settings, in vitro evidence demonstrates that both sealers undergo an initial phase of mild cytotoxicity immediately after mixing due to their high alkalinity and the rapid release of calcium hydroxide during the hydration reaction [25]. Studies by Kumar et al. and Almeida et al. confirmed that this initial cytotoxic effect drops off drastically after the first 24 h as the materials stabilize and begin to form a surface layer of hydroxyapatite, which promotes bioactivity and tissue attachment [25,26]. This specific 24 h stabilization window explains our clinical findings perfectly: the majority of patients who reported pain did so within the first 24 h post-obturation, corresponding directly to the peak chemical activity, high pH, and initial ion release of the freshly extruded materials. Once the 24 h mark passed and the sealers transitioned into their set, bioactive, and cytocompatible states, periapical inflammation subsided rapidly; as a result, the Total Fill group became fully asymptomatic, and the Bio-C group reported minimal pain levels. This demonstrates that the initial in vitro cytotoxicity of these materials represents a self-limiting chemical phase rather than a persistent clinical complication.

Evaluation of patients in the present study also revealed no significant difference in postoperative pain intensity between men and women. Similar findings were reported by Watkins et al., who observed comparable postoperative pain levels between male and female patients following endodontic treatment [27]. In contrast, other studies have reported a higher incidence of postoperative pain in females than in males [5,21,28]. Such variations among studies may be attributed to differences in sample size, age range, gender distribution, treatment protocols, and case selection criteria. In the present investigation, tooth type was not significantly associated with postoperative pain (*p* = 1.00). Similar findings were reported in previous studies by Liddell and Yesilsoy [29,30]. However, these results contrast with those of Ryan et al., who identified female gender and molar teeth as significant factors influencing postoperative pain following endodontic treatment [31].

Beyond the obturation materials evaluated in this trial, the specific parameters of chemomechanical preparation play a critical role in modulating periapical inflammation and subsequent postoperative pain. In this study, mechanical preparation was strictly standardized using the ProTaper Gold system, a decision that directly influences the intersection of instrumentation kinematics, file design, metallurgy, and apical preparation dynamics [32].

While this investigation provides insightful clinical trends, some limitations must be acknowledged. First, the sample size of 15 participants per group, while consistent with the exploratory nature of a pilot trial, increases the possibility of a Type II error; as such, the study was not powered to establish therapeutic equivalence between the two sealers or to detect subtle differences in pain recovery patterns. In addition, the trial was conducted at a single institution (Al-Baha University), which may limit the applicability of the findings to other clinical settings. Furthermore, the strict inclusion criteria and localized sample pool restrict the generalizability of the results to broader patient populations, more complex anatomical presentations, and different operator experience levels. Consequently, the results of this pilot trial should be interpreted as exploratory and hypothesis-generating rather than definitive.

## 5. Conclusions

Within the limitations of this randomized clinical pilot trial, the preliminary data suggest similar short-term clinical performance between Bio-C Sealer and TotalFill BC Sealer in terms of both the incidence and intensity of postoperative pain following non-surgical root canal treatment. While no statistically significant differences in pain levels were observed during the 7-day observational period, these findings must be interpreted with caution given the exploratory nature and limited generalizability of this pilot framework.

## Figures and Tables

**Table 1:** Inclusion and exclusion criteria of eligible patients.

	Criteria
Inclusion	Willing to participate and provide informed consent
	Required root canal treatment
	Medically healthy (ASA Class I or II)
	Aged 18 years or older
	Normal periapical radiographic appearance or a periapical lesion < 2 mm
	Restorable tooth
	Periodontally healthy tooth
Exclusion	Under 18 years of age
	Pregnant
	Declined participation
	Previously endodontically treated tooth
	Tooth with an open apex
	Presence of a deep isolated periodontal pocket
	Tooth fracture
	Root resorption
	Endo-perio lesion
	Non-restorable tooth
	Tooth with mishaps

**Table 2:** Comparison of postoperative pain incidence and intensity between Bio-C Sealer and TotalFill BC Sealer groups at different follow-up intervals.

Follow-Up Intervals		Bio-C Sealer (*n* = 15)	TotalFill BC Sealer (*n* = 15)	Total (*n* = 30)	*p*-Value (Pain Incidence) ^a^	*p*-Value (Mean VAS) ^b^
Post-Op Pain	24 h	5 (33.3%) 1.21 (± 2.21)	4 (26.7%) 0.60 (± 1.12)	9 (30%) 0.9 (± 1.75)	1.000	0.354
	48 h	2 (13.3%) 0.67 (± 2.09)	0 (0%) 0.0 (± 0.00)	2 (6.7%) 0.33 (± 1.49)	0.483	0.241
	72 h	1 (6.7%) 0.53 (± 2.07)	0 (0%) 0.0 (± 0.00)	1 (3.3%) 0.27 (± 1.46)	1.000	0.337
	7 Days	1 (6.7%) 0.13 (± 0.52)	0 (0%) 0.0 (± 0.00)	1 (3.3%) 0.07 (± 0.37)	1.000	0.337

Statistical key: ^a^ Fisher’s exact test. ^b^ Independent *t*-test. Values formatted as count (%) followed by VAS mean (± SD).

## Data Availability

The de-identified datasets generated during the current study are openly available in the Zenodo repository at https://doi.org/10.5281/zenodo.20758377 (accessed on 19 June 2026).

## Supplementary Materials

The following supporting information can be downloaded at: https://www.mdpi.com/article/10.3390/healthcare14152305/s1, Table S1: Incidence of postoperative pain according to sealer type and potential confounding factors (gender and tooth type) at different time intervals following obturation. Table S2: Incidence of postoperative pain according to sealer type and potential confounding factors (sealer puff and pre-operative pain) at different time intervals following obturation.

## Abbreviations

The following abbreviations are used in this manuscript:

VAS	Visual Analogue Scale
SD	Standard Deviation
CONSORT	Consolidated Standards of Reporting Trials

## References

[1] Raja S.N., Carr D.B., Cohen M., Finnerup N.B., Flor H., Gibson S., Keefe F., Mogil J.S., Ringkamp M., Sluka K.A. (2020). The revised IASP definition of pain: Concepts, challenges, and compromises. Pain.

[2] Arslan H., Güven Y., Karataş E., Doğanay E. (2017). Effect of the Simultaneous Working Length Control during Root Canal Preparation on Postoperative Pain. J. Endod..

[3] Patil A.A., Joshi S.B., Bhagwat S.V., Patil S.A. (2016). Incidence of postoperative pain after single visit and two visit root canal therapy: A randomized controlled trial. J. Clin. Diagn. Res..

[4] Seymour R.A. (1982). The use of pain scales in assessing the efficacy of analgesics in post-operative dental pain. Eur. J. Clin. Pharmacol..

[5] Polycarpou N., Ng Y., Canavan D., Moles D.R., Gulabivala K. (2005). Prevalence of persistent pain after endodontic treatment and factors affecting its occurrence in cases with complete radiographic healing. Int. Endod. J..

[6] Genet J., Wesselink P., Van Velzen S.T. (1986). The incidence of preoperative and postoperative pain in endodontic therapy. Int. Endod. J..

[7] Sathorn C., Parashos P., Messer H. (2007). The prevalence of postoperative pain and flare-up in single- and multiple-visit endodontic treatment: A systematic review. Int. Endod. J..

[8] Ali S.G., Mulay S., Palekar A., Sejpal D., Joshi A., Gufran H. (2012). Prevalence of and factors affecting post-obturation pain following single visit root canal treatment in Indian population: A prospective, randomized clinical trial. Contemp. Clin. Dent..

[9] Schilder H. (1967). Filling Root Canals in Three Dimensions. Dent. Clin. N. Am..

[10] Tan H.S.G., Lim K.C., Lui J.N., Lai W.M.C., Yu V.S.H. (2021). Postobturation Pain Associated with Tricalcium Silicate and Resin-based Sealer Techniques: A Randomized Clinical Trial. J. Endod..

[11] Graunaite I., Skucaite N., Lodiene G., Agentiene I., Machiulskiene V. (2018). Effect of Resin-based and Bioceramic Root Canal Sealers on Postoperative Pain: A Split-mouth Randomized Controlled Trial. J. Endod..

[12] Camilleri J. (2020). Classification of Hydraulic Cements Used in Dentistry. Front. Dent. Med..

[13] López-García S., Myong-Hyun B., Lozano A., García-Bernal D., Forner L., Llena C., Guerrero-Gironés J., Murcia L., Rodríguez-Lozano F.J. (2020). Cytocompatibility, bioactivity potential, and ion release of three premixed calcium silicate-based sealers. Clin. Oral Investig..

[14] Chang S.-W., Lee S.-Y., Kang S.-K., Kum K.-Y., Kim E.-C. (2014). In Vitro Biocompatibility, Inflammatory Response, and Osteogenic Potential of 4 Root Canal Sealers: Sealapex, Sankin Apatite Root Sealer, MTA Fillapex, and iRoot SP Root Canal Sealer. J. Endod..

[15] Fonseca B., Coelho M.S., Bueno C.E.d.S., Fontana C.E., De Martin A.S., Rocha D.G.P. (2019). Assessment of Extrusion and Postoperative Pain of a Bioceramic and Resin-Based Root Canal Sealer. Eur. J. Dent..

[16] Zordan-Bronzel C.L., Torres F.F.E., Tanomaru-Filho M., Chávez-Andrade G.M., Bosso-Martelo R., Guerreiro-Tanomaru J.M. (2019). Evaluation of Physicochemical Properties of a New Calcium Silicate–based Sealer, Bio-C Sealer. J. Endod..

[17] Fink R. (2000). Pain Assessment: The Cornerstone to Optimal Pain Management. Bayl. Univ. Med. Cent. Proc..

[18] Omoigui S. (2007). The biochemical origin of pain: The origin of all pain is inflammation and the inflammatory response. Part 2 of 3—Inflammatory profile of pain syndromes. Med. Hypotheses.

[19] Delgado D.A., Lambert B.S., Boutris N., McCulloch P.C., Robbins A.B., Moreno M.R., Harris J.D. (2018). Validation of Digital Visual Analog Scale Pain Scoring With a Traditional Paper-based Visual Analog Scale in Adults. JAAOS Glob. Res. Rev..

[20] Ng Y.L., Glennon J.P., Setchell D.J., Gulabivala K. (2004). Prevalence of and factors affecting post-obturation pain in patients undergoing root canal treatment. Int. Endod. J..

[21] Pak J.G., White S.N. (2011). Pain Prevalence and Severity before, during, and after Root Canal Treatment: A Systematic Review. J. Endod..

[22] Yu V.S.H., Messer H.H., Yee R., Shen L. (2012). Incidence and Impact of Painful Exacerbations in a Cohort with Post-treatment Persistent Endodontic Lesions. J. Endod..

[23] Segura-Egea J.J., Cisneros-Cabello R., Llamas-Carreras J.M., Velasco-Ortega E. (2009). Pain associated with root canal treatment. Int. Endod. J..

[24] De-Figueiredo F.E.D., Lima L.F., Lima G.S., Oliveira L.S., Ribeiro M.A., Brito-Junior M., Correa M.B., Sousa-Neto M.D., Faria e Silva A.L. (2020). Apical periodontitis healing and postoperative pain following endodontic treatment with a reciprocating single-file, single-cone approach: A randomized controlled pragmatic clinical trial. PLoS ONE.

[25] Kour S., Kumar A., Kaul S., Malik A., Dhani R., Kaul R. (2023). Cytotoxicity evaluation of Bio-C, CeraSeal, MTA—Fillapex, and AH Plus root canal sealers by microscopic and 3-(4, 5 dimethythiazol-2yl)-2, 5-diphenyltetrazolium bromide (MTT) assay. J. Conserv. Dent..

[26] Almeida M., Rodrigues C., Matos A., Carvalho K., Silva E., Duarte M., Oliveira R., Bernardineli N. (2020). Analysis of the physicochemical properties, cytotoxicity and volumetric changes of AH Plus, MTA Fillapex and TotalFill BC Sealer. J. Clin. Exp. Dent..

[27] Watkins C.A., Logan H.L., Kirchner H.L. (2002). Anticipated and experienced pain associated with endodontic therapy. J. Am. Dent. Assoc..

[28] Sadaf D., Ahmad M.Z. (2014). Factors associated with postoperative pain in endodontic therapy. Int. J. Biomed. Sci..

[29] Liddell A., Locker D. (1997). Gender and age differences in attitudes to dental pain and dental control. Community Dent. Oral Epidemiol..

[30] Yesilsoy C., Koren L.Z., Morse D.R., Rankow H., Bolanos O.R., Furst M.L. (1988). Post-endodontic obturation pain: A comparative evaluation. Quintessence Int..

[31] Ryan J.L., Jureidini B., Hodges J.S., Baisden M., Swift J.Q., Bowles W.R. (2008). Gender Differences in Analgesia for Endodontic Pain. J. Endod..

[32] Abed K.H., Hashem A.A.R., Lutfy R.A., Eissa S.A., Morsy D.A. (2026). Effect of different rotary instrument designs (protaper ultimate and protaper gold) on postoperative pain and bacterial reduction: A randomized clinical trial. BMC Oral Health.

