# Supplementary material for: Postoperative Pain After Root Canal Filling with Different Bioceramic Endodontic Sealers: A Double-Blind Randomized Clinical Pilot Trial
Source: Healthcare (Basel). 2026 Jul 31;14(15):2305. doi: 10.3390/healthcare14152305 (PMC13465557; doi:10.3390/healthcare14152305)
Supplement: Supplementary file 1 [file healthcare-14-02305-s001.zip › Tables S1 and S2.pdf]

Table S1. Incidence of postoperative pain according to sealer type and potential confounding factors (gender and tooth type) at different time intervals following obturation.

Sealer Type	Subgroup	Postoperative pain				*p*-value (24 h)	*p*-value (48 h–7 d)
		**24 h**	**48 h**	**72 h**	**7 Days**		
Bio-C Sealer	Male (*n* = 15)	3 (20%)	0 (0%)	0 (0%)	0 (0%)	0.23	1.000
	Female (*n* = 15)	2(13.3%)	2(13.3%)	1(6.7%)	1(6.7%)		
TotalFill BC Sealer	Male (*n* = 15)	1 (6.7%)	0 (0%)	0 (0%)	0 (0%)	0.51	*N*/A
	Female (*n* = 15)	3(20.0%)	0 (0%)	0 (0%)	0 (0%)		
Bio-C Sealer	Molar (*n* = 10)	4 (40%)	2 (20%)	1(10%)	1(10%)	1.00	0.524
	Non-Molar Molar (*n* = 5)	1 (20%)	0 (0%)	0 (0%)	0 (0%)		
TotalFill BC Sealer	Molar (*n* = 9)	2(22.2%)	0 (0%)	0 (0%)	0 (0%)	1.00	*N*/A
	Non-Molar (*n* = 6)	2(33.3%)	0 (0%)	0 (0%)	0 (0%)		

Table S2. Incidence of postoperative pain according to sealer type and potential confounding factors (sealer puff and pre-operative pain) at different time intervals following obturation.

Sealer Type	Sub-group	Post-operative pain				*p*-value (24 h)	*p*-value (48 h-7d)
		**24 h**	**48 h**	**72 h**	**7 Days**		
Bio-C Sealer	Puff (+) (*n* = 11)	5 (45%)	2 (18%)	1 (9%)	1 (9%)	0.23	1.00
	Puff (–) (*n* = 4)	0 (0%)	0 (0%)	0 (0%)	0 (0%)		
TotalFill BC Sealer	Puff (+) (*n* = 11)	2 (18%)	0 (0%)	0 (0%)	0 (0%)	0.51	*N*/A
	Puff (–) (*n* = 4)	2 (50%)	0 (0%)	0 (0%)	0 (0%)		
Bio-C Sealer	Pre-op Pain (+) (*n* = 5)	1 (20%)	0 (0%)	0 (0%)	0 (0%)	1.00	1.00
	Pre-op Pain (–) (*n* = 10)	4 (40%)	2 (20%)	1(10%)	1(10%)		
TotalFill BC Sealer	Pre-op Pain (+) (*n* = 3)	1 (33%)	0 (0%)	0 (0%)	0 (0%)	0.47	*N*/A
	Pre-op Pain (–) (*n* = 12)	3 (25%)	0 (0%)	0 (0%)	0 (0%)		
Patients Requiring Analgesics (*n*,%)	Bio-C Sealer	5(33.3%)				1.000	N/A
	TotalFill BC Sealer	4(26.7%)					
